# How to Deal with the Telephone Sales Menace

**Published:** 1990-03

**Authors:** J. L Burton


### HOW TO DEAL WITH THE TELEPHONE SALES MENACE

Our household seems to be receiving more and more telephone calls from persons unknown trying to sell anything from insurance to double glazing. The calls are carefully timed to coincide with the evening meal, and the chagrin this causes is considerable. Doctors are particularly vulnerable to this annoyance, as they can't take the phone off the hook, and its particularly frustrating that one is unable to convey the depth of one's feelings to the caller without using words you wouldn't want the children to overhear.

I have recently developed a technique for dealing with this menace which I recommend for its therapeutic value. It saves no time, but if you've a few minutes to spare while you're sipping a sherry and waiting for the potatoes to cook, it can be a very satisfying experience.

The technique can be divided into 3 stages. In the first stage, one shows a keen interest in the product, and strings the salesman along for as long as possible, at his expense of course. In the next stage, one graudally introduces obstacles of progressively increasing difficulty which he has to surmount. Only at your convenience do you allow him suddenly to realize that he is dealing with a raving lunatic and he has toally wasted his time and money. The sense of power this produces is sublime, and is particularly soothing for overworked doctors who have been treated like trainee filing-clerks all day.

A sample conversation would go as follows:

*Stage 1. The bait is taken.*

*A Super Salesman (ASS):* Good evening sir, I'm from the Bloggs Corporation. I expect you're aware how much house prices have shot up in the last year or two, but you might not know just how much damage acid rain can do to the fabric of your house. We are doing a survey in your district at present to try and find out how much damage is being done, and to see how many houses have adequate exterior protection against this type of damage.

*Gullible Overworked Doctor (GOD):* Oh really, that sound interesting, what's it all about then?

ASS then launches into sales pitch, and GOD makes interested noises from time to time, interspersed with intelligent questions. It is important at this stage to convey that you are well-heeled, and a huge sale is a distinct possibility.

At length ASS feels he's done enough and moves in for the kill.

*Stage 2. The struggle.*

*ASS:* Perhaps I could make an appointment then for our Mr. Snigglethorpe to call and give you an estimate?

*GOD:* Yes, that would be very nice, when can he call?

*ASS:* Any time at your convenience sir

*GOD:* Oh wonderful. Well what's the best day for him?

*ASS:* What about next Wednesday or Thursday?

*GOD:* Well now let me see . . . next week's out because of the soirée at the Royal Society, then I'm away in Paris for 2 weeks, and then I'm back for 3 days, but I'll be getting ready for my Australia trip, so I think it'll have to be when I get back from Australia, would that be alright?

*ASS:* Yes, perfect, when will that be then?

*GOD:* Well its going to be August I'm afraid.

*ASS:* Oh dear, I don't know if I can make appointments that far ahead . . . wait a moment while I check with my supervisor . . . long pause) . . . Yes that's O.K. . . . now let me see if I can just find next year's calendar . . . (another long pause) . . . ah here it is, now what date shall we say?

*GOD:* How about Wednesday 12th?

*ASS:* I think you're mistaken sir, the 12th of August is a Friday

*GOD:* Oh sorry, you're loking at the 1990 dairy are you? No I'm not back from Australia for 3 years . . . that's Wednesday 12th, 1992 I'm talking about.

*ASS:* (Another long pause) Now, I'm sorry sir but we just can't book appointments that far ahead.

*GOD:* Well why not? You'll still be in business in 3 years time won't you? Suppose you come and put your protective paint on my house this year and it all peels off in 2 years time, are you trying to tell me your firm will have folded up by then?

*ASS:* Certainly not sir, we expect to be in business in 30 years from now, never mind 3 years.

*GOD:* So why can't you make an appointment for me to have my house seen in 3 years time?

*ASS:* Well I suppose the main reason is we don't have a calendar that goes that far ahead

*GOD:* That's an insurmountable problem for a firm with your technological expertise is it? Or is it the capital outlay that's the problem? Anyway I'm telling you that August 12th will be a Wednesday. Could I speak to your Supervisor for a minute if you can't handle this?

*ASS:* Just a minute sir . . . (another long pause, with muttered conversation in the background) . . .

And so on and so on, until the potatoes are cooked or your glass is empty and then . . .

*Stage 3. The kill*

*ASS:* (Wearly) Well, what sort of outer cladding do you have on the walls of your house at the moment sir?

*GOD:* Fur.

*ASS:* (Further pause, then in ice-cold tones) I see, thank you very much sir.

*GOD:* My pleasure I assure you. Goodbye.

J. L BURTON

